# Association between loss of Y chromosome and poor prognosis in male head and neck squamous cell carcinoma

**DOI:** 10.1002/hed.25537

**Published:** 2018-12-23

**Authors:** Robert Hollows, Wenbin Wei, Jean‐Baptiste Cazier, Hisham Mehanna, Gabriella Parry, Graham Halford, Paul Murray

**Affiliations:** ^1^ Institute of Cancer and Genomic Sciences University of Birmingham Birmingham United Kingdom; ^2^ Centre for Computational Biology University of Birmingham Birmingham United Kingdom; ^3^ Sheffield Institute of Translational Neuroscience University of Sheffield Sheffield United Kingdom; ^4^ Institute of Head and Neck Studies and Education (InHANSE) University of Birmingham Birmingham United Kingdom; ^5^ Regional Genetics Laboratory Birmingham Women's Hospital Birmingham United Kingdom

**Keywords:** aneuploidy, head and neck cancer, immune system, therapeutic resistance, Y chromosome

## Abstract

**Background:**

Head and neck squamous cell carcinoma (HNSCC) is more prevalent in men than women and this disparity cannot be fully explained by known risk factors. Recent studies have shown that loss of Y chromosome (LoY) confers an increased risk of solid cancer and reduces life expectancy in men.

**Methods:**

Using publicly available data from The Cancer Genome Atlas, we investigated the prevalence of LoY and its association with clinicopathological features in male HNSCC.

**Results:**

LoY was detectable in around 25% of male HNSCC. Men with human papillomavirus‐negative tumors exhibiting LoY experienced significantly worse overall survival than those with no LoY. Moreover, LoY tumors exhibited overexpression of genes involved in redox processes, including genes previously implicated in resistance to both radiotherapy and cisplatin‐based chemotherapeutics.

**Conclusion:**

LoY may be an indicator of poor prognosis in male HNSCC that is linked to the overexpression of genes associated with resistance to standard care therapies.

## 1 INTRODUCTION

Head and neck cancer is the sixth most common cancer worldwide^1^ and can be categorized by anatomic subsite into tumors of the oropharynx, nasopharynx, hypopharynx, oral cavity, and larynx.^2^ Head and neck squamous cell carcinoma (HNSCC) is the most common histological subtype, with around six hundred thousand new cases worldwide each year.^2^ The management of HNSCC consists mainly of multiple‐modality therapy with surgery, radiation, and chemotherapy. However, despite significant improvements in these therapies, long‐term survival rates in patients with advanced‐stage HNSCC have not increased significantly in the past 30 years, remaining around only 50% at 5 years.^3^

Smoking and alcohol consumption are well‐established risk factors for HNSCC, and may act synergistically during tumor initiation.^3^, ^4^ Although HNSCC exhibits considerable genetic heterogeneity, mutations in the TP53 gene and chromosomal instability are common features.^3^ In recent years, infection with the human papillomavirus (HPV) has been identified as a causative agent in around 25% of cases.^2^, ^5^ HPV‐positive cases mostly arise in the oropharynx,^2^ and patients with these tumors are reported to have a better outcome than patients with HPV‐negative tumors.^6^ Although the incidence of HPV‐negative cancers appears to be declining as smoking becomes less common, HPV‐positive cancers have become more prevalent in recent years.^7^

Head and neck cancer is more prevalent in men than women.^1^ Such sex imbalance is characteristic of many cancers, and it is not adequately explained by differences in exposure to key risk factors.^8^ This unexplained disparity raises the possibility that underlying genetic differences between men and women may make the former more susceptible to certain cancers, including tumors of the head and neck.

The most obvious genetic difference between the sexes is that females have two X chromosomes, whereas males have only one, maternally derived, X chromosome, and one Y chromosome. Unlike the autosomes, the X and Y chromosomes only recombine in two short regions at the tips of either arm, called the pseudoautosomal regions (PARs). The vast majority of the Y chromosome between the PARs is referred to as the male‐specific region. The Y chromosome is the third shortest chromosome, and contains only a small complement of functional genes as a result of millions of years of gene‐decay since the mammalian sex‐chromosomes first evolved from a pair of ancestral autosomes.^9^ However, there is mounting evidence that despite the relative paucity of genes, the Y chromosome is critically important for biological functions beyond its role in male sex determination.^10^, ^11^ In particular, loss of Y chromosome (LoY) is implicated in cancer. For example, recent studies of over 1000 elderly men showed that mosaic LoY in blood cells is associated with smoking, an increased risk of non‐hematological cancer and impaired life expectancy.^12^, ^13^

Although LoY has been documented in HNSCC,^14^ to date no studies have reported its significance, either in terms of the underlying biology or clinical impact. Here, we report our findings on the prevalence of LoY in male HNSCC, and its association with clinicopathological features, based on analyses of publicly available data from The Cancer Genome Atlas (TCGA).^15^

## 2 MATERIALS AND METHODS

### 2.1 Datasets used

The primary data used was TCGA's HNSC dataset. This comprised 369 male and 135 female tumor samples.

The secondary dataset used comprised gene expression data for 167 oral tumor samples (120 male), 17 oral dysplasia samples (10 male), and 45 normal oral samples (32 male) reported by Chen et al.^16^

For fluorescence in situ hybridization (FISH) analysis, sections of a tissue microarray (TMA) containing oral, oropharyngeal, hypopharyngeal, and laryngeal tumors were obtained under ethical approval (10/H1210/9). These comprised, in particular, 27 male tumor samples.

### 2.2 Copy number and mutation analyses using TCGA data

Analyses of copy number variation were based on TCGA's level 3 segmented copy number data produced using the Affymetrix Genome‐Wide Human SNP Array 6.0. These data were downloaded from TCGA's data portal on February 5, 2015. We also downloaded clinical data in the “Biotab” format and TCGA's level 2 somatic mutation data that had been produced using the IlluminaGA DNASeq platform.

We used the copy number data based on version hg19 of the human reference genome. For each sample, the segmented data were used to calculate an average copy number “index” for each chromosome and chromosome arm, as the average copy number across the whole length of the chromosome/arm. The results were adjusted for tumor purity using the TCGA clinical data item “tumor_nuclei_percent.”

To calculate the total autosomal aneuploidy index, for each sample we summed the absolute differences between two and the copy number index for each autosomal arm. Data were not available for the short arms of the acrocentric chromosomes 13, 14, 15, and 22, so these arms were excluded from our analyses.

### 2.3 Differential expression analyses

TCGA's level 3 RNA‐sequencing data based on the Illumina HiSeq 2000 RNA Sequencing (Version 2) platform were downloaded. We used the files labeled “rsem.genes.results,” which contained un‐normalized read counts for over 20 000 genes. Read counts were normalized between samples and converted to counts‐per‐million (cpm) reads for each gene using the edgeR package^17^ in R.^18^ The same package was used to perform differential expression analysis, using the following criteria: fold‐change ± 1.5, *P* < 0.05 (or <0.01 for HPV‐negative cases), and cpm > 1 in at least the number of samples in the smaller comparison group.

The raw CEL files used by Chen et al^16^ were downloaded and reanalyzed using probe level quantile normalization,^19^ robust multi‐array analysis,^20^ and mas5 detection analysis. The “affy” package in R was used for this purpose. Seven of 27 Y chromosome probes (“201909_at” gene = RPS4Y1, “230760_at” [ZFY], “228482_at” [USP9Y], “205000_at” [DDX3Y], “236694_at” [CYorf15A], “206700_s_at” [KDM5D], and “204409_s_at” [EIF1AY]) were found to differentiate male and female samples. While analyzing the data we noticed that the sex of three tumor samples (two males and one female) were inconsistent with the expression data and were therefore changed.

For each of the seven probes, we calculated an expression ratio for each male sample as follows: (1) the average female expression value across all female samples (assumed to reflect “background” measurement) was calculated; (2) the average female value was deducted from the expression value for each male sample; and (3) the female‐adjusted expression value was divided by the average female‐adjusted expression value for all male normal samples. Principal components analysis was performed on the expression ratios to identify cases with LoY, reasoning that combining measurements would reduce the impact of variability in the measurements for individual probes. The “LIMMA” package in R was used for differential expression analysis, using the following criteria: fold‐change ± 1.5, *P* < 0.01, presence call of “P” in at least the number of samples in the smaller comparison group.

Gene ontology analysis of differentially expressed genes was performed using the Functional Annotation facility of David (v6.8) and GOTERM_BP_DIRECT.^21^, ^22^

### 2.4 Statistical tests

All calculations were performed using R. The “Survival” package was used for survival analyses. Comparisons of LoY and aneuploidy levels between sample groups were performed using the Kruskal‐Wallis test. Correlation analyses were performed using Spearman's method. Unless specified otherwise, a significance level of 5% was used in all statistical tests.

### 2.5 FISH analysis

TMA sections were deparaffinized in two changes of UltraClear (AVANTOR) each of 6 minutes followed by rehydration in two changes of 100% methanol each of 5 minutes. Slides were immersed in 0.2 M HCl for 23 minutes and washed in distilled water for 2 minutes. Spotlight paraffin pretreatment solution (Invitrogen) was heated to 95°C in a water bath prior to immersing slides for 90 minutes, followed by two washes in distilled water each of 2 minutes. Three drops of enzyme digestion solution were added and contained with a coverslip secured by a rubber sealant. Slides were transferred to a humidified chamber at 37°C for 60 minutes, coverslips were removed and slides washed twice in distilled water for 2 minutes and then dehydrated in methanol. Probe solutions for SRY (Vysis), XYq telomere (Cytocell), and XYq (Cytocell) were labeled with fluorescein isothiocyanate (FITC) and tetramethylrhodamine isothiocyanate (TRITC) and hybridized to TMA sections on the HYbrite hybridization hot plate at 73°C (Vysis)/75°C (Cytocell) for 2 minutes followed by 37°C for 16 hours. After hybridization, slides were washed in 0.4xSSC/0.3% Nonidet‐P40 and then in 2xSSC/0.1% Nonidet‐P40 preheated to 73°C in a water bath for 2 minutes and 30 seconds, respectively. The slides were air dried in the dark for 30 seconds and then counter stained with 4′,6‐diamidino‐2‐phenylindole (DAPI), mounted with a coverslip and kept in the dark to protect the integrity of the probe. Signals were recorded using the Olympus BX50 fluorescence microscope at ×1000 magnification. Images were acquired using the Metasystems CoolCube 1 and were analyzed using MetaSystem Isis software (MetaSystem).

## 3 RESULTS

### 3.1 LoY in a subset of males with HNSCC

We used TCGA's segmented copy number data to investigate LoY in 369 male HNSCC. The data covered four of the five main anatomical regions (Table 1). The most commonly affected site was the oral cavity. HPV status was available for 368 cases, of which 85 were classified as HPV‐positive.

**Table 1:** Summary of The Cancer Genome Atlas (TCGA) male head and neck squamous cell carcinoma (HNSCC) data

Site	HPV‐negative	HPV‐positive	Total
Hypopharynx	1 (0.4%)	5 (5.9%)	6 (1.6%)
Oropharynx	20 (7.0%)	49 (57.6%)	69 (18.8%)
Larynx	88 (31.1%)	6 (7.1%)	94 (25.5%)
Oral cavity	174 (61.5%)	25 (29.4%)	199 (54.1%)
Total	283	85	368

Abbreviation: HPV, human papillomavirus.

**Figure 1:**
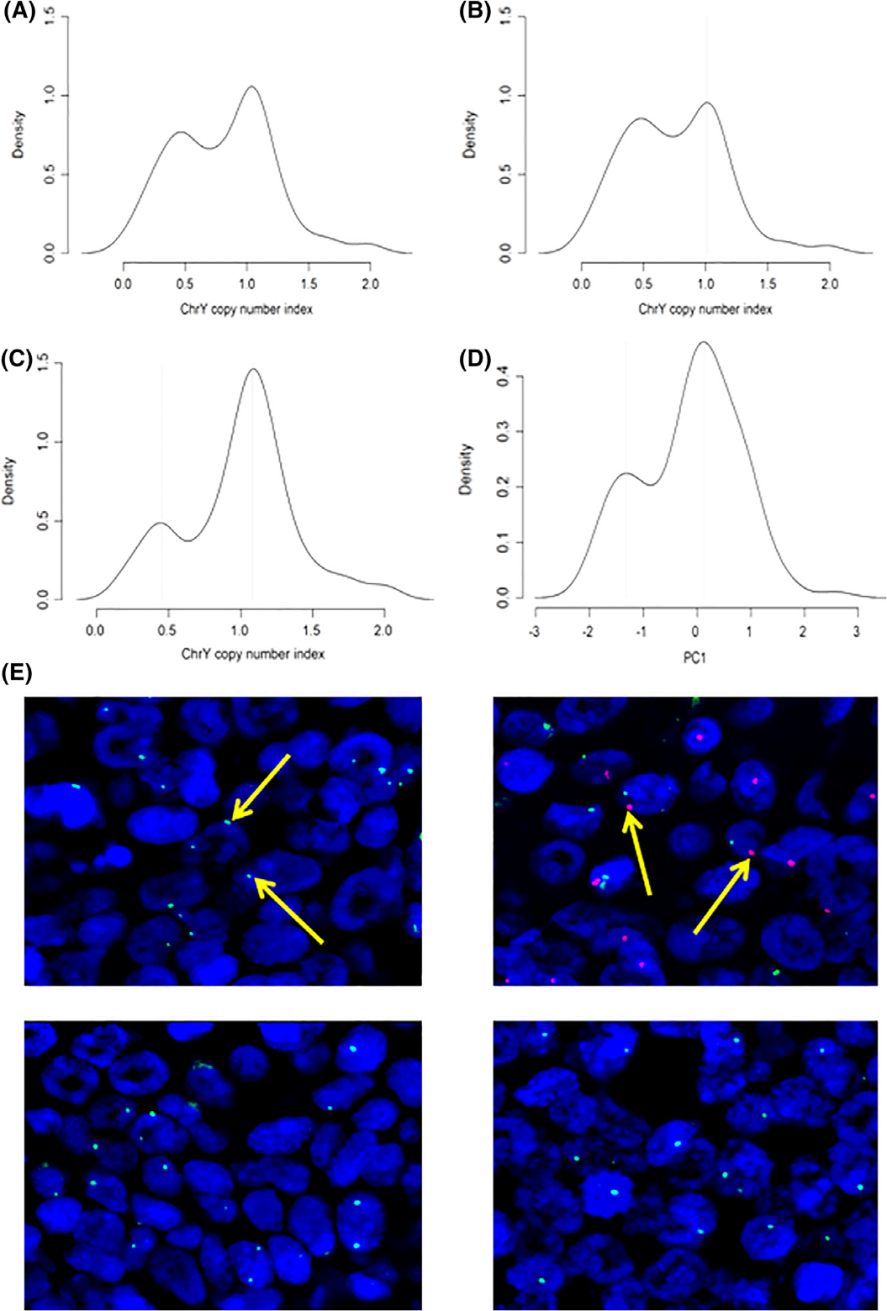
Loss of Y chromosome (LoY) in male head and neck squamous cell carcinoma (HNSCC). A, Density plot of Y chromosome copy number index values for 369 male HNSCC. B,C, Density plots of Y chromosome copy number index values for 283 human papillomavirus (HPV)‐negative and 85 HPV‐positive male HNSCC, respectively. D, Density plot of first principal component values for male oral cavity tumor cases from Chen et al. E, Representative images of fluorescence in situ hybridization (FISH) analysis—X and Y chromosome probes shown in green and red, respectively: top left, female sample; top right, male sample which has retained the Y chromosome; bottom panels, two male samples which have lost the Y chromosome [Color figure can be viewed at wileyonlinelibrary.com]

To measure LoY, we derived a Y chromosome copy number index value for each sample based on the average copy number across the whole male‐specific region (the data did not cover the PARs). This analysis revealed a bimodal distribution of Y chromosome copy number index values for the male tumor samples, with one peak at around 0.5, representing cases which had lost the Y chromosome, and a second higher peak at around 1.0, representing cases which had not lost the Y chromosome (Figure 1A). The bimodal distribution was evident for both HPV‐negative and HPV‐positive tumors when analyzed separately, but LoY was more common in HPV‐negative cases (Figure 1B,C). For HPV‐negative cases, the peaks in the distribution occurred at 0.48 and 1.01. There were 81 (28.6%) cases to the left of the first peak, 124 (43.8%) between the two peaks, and 78 (27.6%) to the right of the second peak. For HPV‐positive cases, the two peaks occurred at 0.45 and 1.08, and the corresponding breakdown of samples was 10 (11.8%) to the left of the first peak, 38 (44.7%) between the two peaks, and 37 (43.5%) to the right of the second peak.

Loss was mostly of the whole Y chromosome, although there were some cases in which the long arm was lost but the short arm was not lost (data not shown). We used the RNA‐seq data available on the same patients to identify genes whose expression was correlated with the Y chromosome index. We identified 19 genes, all on the Y chromosome, for which there was a Spearman correlation of more than 0.5 between gene expression and the Y chromosome index (Table 2). These included all 12 genes previously reported to be critical for male viability.^10^

**Table 2:** Genes whose expression is highly correlated with Y chromosome copy number index

Gene	Correlation	Location
KDM5D^a^	0.85	Long arm
CYorf15A^a^	0.84	Long arm
DDX3Y^a^	0.83	Long arm
UTY^a^	0.82	Long arm
TTTY15	0.82	Long arm
EIF1AY^a^	0.81	Long arm
USP9Y^a^	0.80	Long arm
TMSB4Y^a^	0.78	Long arm
CYorf15B	0.75	Long arm
NLGN4Y^a^	0.73	Long arm
PRKY^a^	0.70	Short arm
ZFY^a^	0.66	Short arm
NCRNA00185	0.66	Long arm
RPS4Y1^a^	0.64	Short arm
NCRNA00230B	0.63	Long arm
TTTY14	0.58	Long arm
SFRS17A	0.56	PAR1 – Short arm
SRY	0.53	Short arm
TBL1Y^a^	0.53	Short arm

Included in Bellott et al.^10^

Sufficient numbers of other male HNSCC with copy number data were not available in the published literature. Therefore, to confirm our observations of LoY in a separate cohort, we took advantage of the global gene expression data available on 119 male oral cancer samples reported by Chen et al.^16^ Using principal components analysis based on the expression ratios for the seven most discriminating probes, we identified a subset of male cases with reduced expression consistent with LoY. For the 119 tumor cases, a density plot of the first principal component values revealed a bimodal distribution similar to that found in the TCGA data (Figure 1D).

Finally, to provide unequivocal evidence of LoY in male HNSCC, we performed FISH on a separate cohort of 27 male HNSCC using probes to detect the centromere of the Y chromosome. Consistent with the data presented above, we observed LoY in the tumor cells of 6 of 27 male HNSCC samples (Figure 1E).

We conclude that around one‐quarter of male HNSCC are characterized by LoY.

### 3.2 LoY may be linked to smoking in men with HPV‐negative HNSCC

LoY has previously been reported to be associated with both smoking and increasing age.^13^, ^23^

Data on smoking history at diagnosis (categorized as “never smoked,” “stopped smoking more than 15 years ago,” “stopped smoking within the last 15 years,” and “current smoker”) were available for 359 TCGA patients with known HPV status (Table 3).

**Table 3:** Breakdown of The Cancer Genome Atlas (TCGA) male head and neck squamous cell carcinoma (HNSCC) data by smoking history and TP53 mutation status

	HPV‐negative			HPV‐positive		
Category	Mutated	Wild type	N/A	Mutated	Wild type	N/A
Never smoked	36 (87.8%)	5 (12.2%)	0 (0.0%)	3 (12.5%)	21 (87.5%)	0 (0.0%)
Stopped >15 y	25 (65.8%)	11 (28.9%)	2 (5.3%)	3 (25.0%)	9 (75.0%)	0 (0.0%)
Stopped <15 y	69 (84.1%)	11 (13.4%)	2 (2.4%)	6 (24.0%)	17 (68.0%)	2 (8.0%)
Current smoker	93 (81.6%)	14 (12.3%)	7 (6.1%)	9 (39.1%)	13 (56.5%)	1 (4.3%)
Total	223 (81.1%)	41 (14.9%)	11 (4.0%)	21 (25.0%)	60 (71.4%)	3 (3.6%)

Abbreviation: HPV, human papillomavirus.

**Figure 2:**
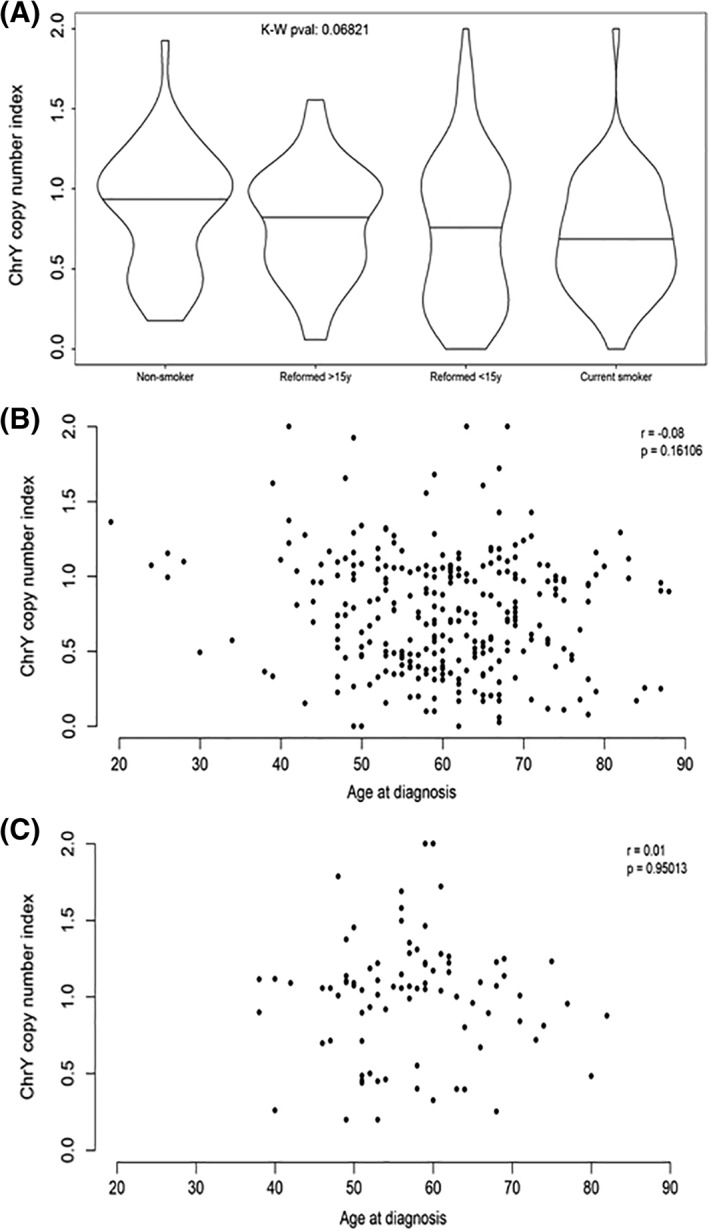
Loss of Y chromosome (LoY) may be associated with smoking history but not age for human papillomavirus (HPV)‐negative male head and neck squamous cell carcinoma (HNSCC). A, Violin plots of Y chromosome copy number index values by smoking history for HPV‐negative male HNSCC—horizontal lines denote median values. B,C, Plots of Y chromosome copy number index values against age at diagnosis for HPV‐negative and HPV‐positive male HNSCC, respectively

For the HPV‐negative cases, we found that the Y chromosome index decreased across the smoking categories from nonsmoker to current smoker, although this association was only of borderline significance (Figure 2A, Kruskal‐Wallis *P* = 0.07). No such pattern was observed for the HPV‐positive cases (data not shown, Kruskal‐Wallis *P* = 0.61). We observed no statistically significant association between age and LoY for either HPV subgroup (Figure 2B,C).

We conclude that LoY is not correlated with age, but may be linked to smoking in men with HPV‐negative HNSCC.

### 3.3 LoY is associated with shorter overall survival in HPV‐negative male HNSCC

We next investigated if LoY was associated with overall survival in men with HNSCC.

We first performed a univariate analysis of overall survival separately for males with HPV‐negative or HPV‐positive cancer. For both groups, we compared samples in which the Y chromosome index value fell outside the two peaks of the bimodal distribution, thereby excluding cases that lay between the two peaks and for which we were less confident of LoY status.

**Figure 3:**
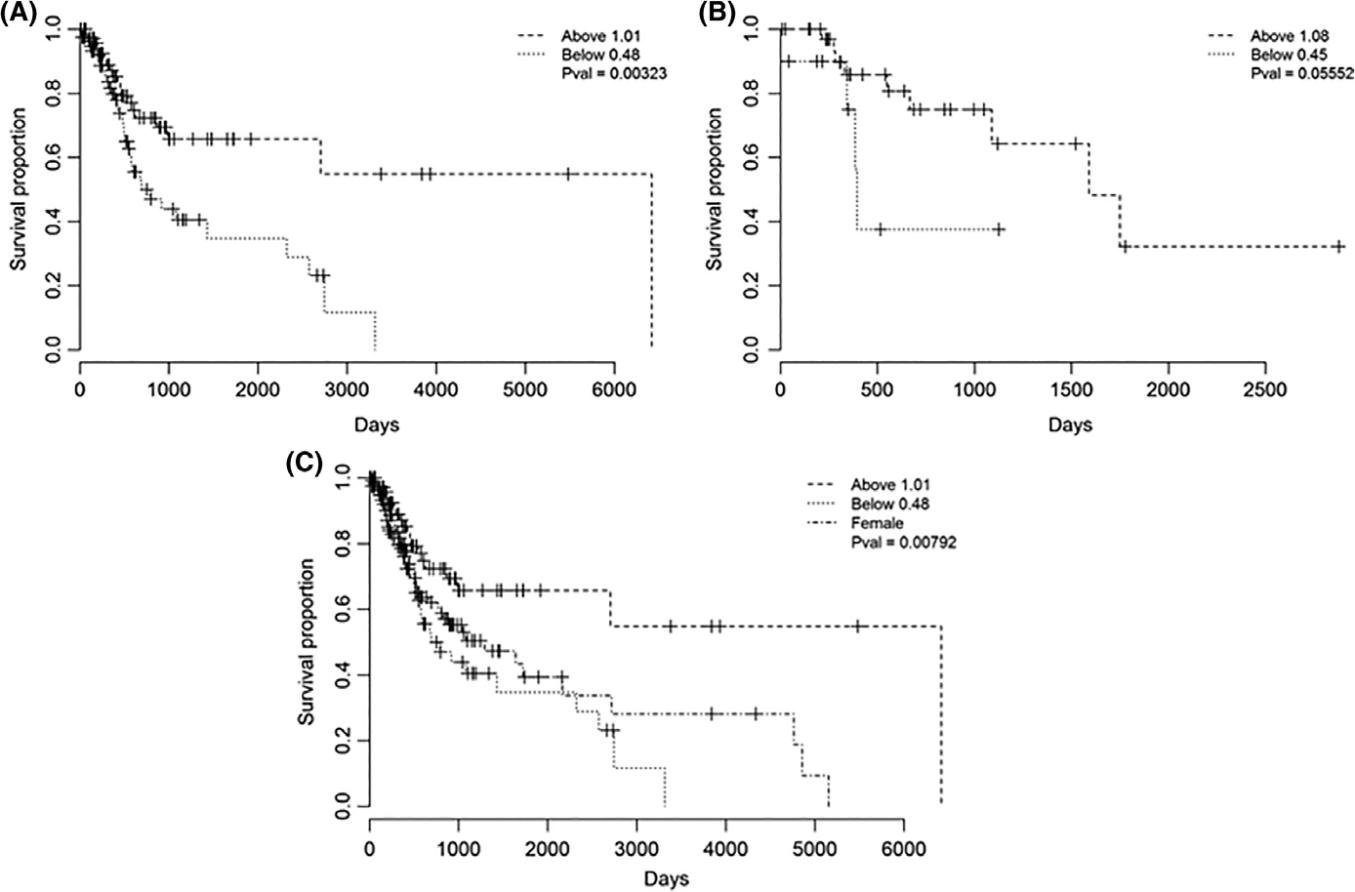
Loss of Y chromosome (LoY) is associated with shorter overall survival in male head and neck squamous cell carcinoma (HNSCC). Kaplan‐Meier plots of overall survival for male HNSCC in A, human papillomavirus (HPV)‐negative cases split by LoY status; B, HPV‐positive cases split by LoY status; and C, HPV‐negative cases split by LoY status and compared to HPV‐negative females with HNSCC

We found that for HPV‐negative HNSCC, LoY cases had significantly shorter overall survival than non‐LoY cases, whereas for HPV‐positive patients there was a similar trend, but this was marginally not statistically significant (Figure 3A,B, log‐rank *P* = 0.003 and 0.06, respectively).

We next performed separate univariate survival analyses for the same HPV‐negative cases using each of the clinical items shown in Table 4. We found that pathologic N had the most statistically significant association with overall survival (*P* = 0.002), closely followed by LoY status (*P* = 0.003) and pathologic T (*P* = 0.006).

**Table 4:** Summary of univariate survival analyses for human papillomavirus (HPV)‐negative male head and neck squamous cell carcinoma (HNSCC) patients

Item	Number of patients for which data available	Categories used for Kaplan‐Meier analysis	*P* value
Pathologic N	139	N0/N1/N2/N3	0.002
LoY status	159	Y index <0.48 vs > 1.01	0.003
Pathologic T	146	T1/T2/T3/T4/T4a/T4b	0.006
Perineural invasion	119	Yes / no	0.02
Pathologic stage	145	Stage I/II/III/IV	0.18
Histologic grade	153	G1/G2/G3/G4	0.49
TP53 mutation	155	Yes / no	0.31
Age (median split)	159	< median / > median	0.21
Smoking history	153	As described in text	0.94
Anatomic site	159	Oral cavity / larynx / oropharynx	0.92

Abbreviation: LoY, loss of Y chromosome.

Univariate Cox proportional hazards analysis of all HPV‐negative patients using the Y chromosome index value confirmed the association with overall survival (Wald test *P* = 0.02). However, the Y chromosome index was no longer significantly associated with survival when considered in a multivariate analysis with pathologic N, but did retain a borderline significant association (Wald test *P* = 0.05) when considered together with pathologic T. There was a clear association between lower Y chromosome index and higher pathologic T category (data not shown, Kruskal‐Wallis *P* = 0.0002).

Finally, we compared overall survival in males and females with HPV‐negative HNSCC. We found that HPV‐negative females with HNSCC had significantly longer overall survival than male LoY cases, but significantly worse overall survival than non‐LoY cases (Figure 3C, *P* = 0.008).

We conclude that LoY is associated with significantly shorter overall survival in males with HPV‐negative HNSCC.

### 3.4 LoY is associated with reduced expression of immune genes and overexpression of redox‐related genes in male HNSCC

To determine if cases with LoY define a phenotypically distinct subset of male HNSCC and to identify genes that could potentially explain the association between LoY and poorer patient outcomes, we compared global gene expression in cases with or without LoY. For each HPV subgroup, we again compared cases for which the Y chromosome index value fell outside the two peaks of the bimodal distribution.

We found that genes downregulated in LoY cases compared with non‐LoY cases were enriched for ontology terms associated with immune‐related functions, including “immune response,” “adaptive immune response,” and “inflammatory response.” Genes upregulated in this comparison were enriched for genes with functions in “oxidation‐reduction process” (redox). These effects were evident in both HPV subgroups (Tables 5 and 6).

**Table 5:** Genes differentially expressed in loss of Y chromosome (LoY) cases for The Cancer Genome Atlas (TCGA) human papillomavirus (HPV)‐negative male head and neck squamous cell carcinoma (HNSCC)

Top 10 most significant gene ontology terms			
Term	No. genes	Fold enrichment	*P* value
**Downregulated genes in LoY cases**			
GO:0006955∼immune response	94	4.89	1.51E‐38
GO:0006954∼inflammatory response	77	4.45	1.23E‐28
GO:0002250∼adaptive immune response	38	5.62	7.8E‐18
GO:0070098∼chemokine‐mediated signaling pathway	25	7.71	3.27E‐15
GO:0031295∼T cell costimulation	26	7.3	3.53E‐15
GO:0007155∼cell adhesion	64	3.05	3.64E‐15
GO:0060333∼interferon‐gamma‐mediated signaling pathway	24	7.4	3.48E‐14
GO:0007165∼signal transduction	112	2.11	4.13E‐14
GO:0006935∼chemotaxis	30	5.38	1.33E‐13
GO:0002504∼antigen processing and presentation of peptide or polysaccharide antigen via MHC class II	13	16.74	3.74E‐13
**Upregulated genes in LoY cases**			
GO:0055114∼oxidation‐reduction process	40	2.88	5.92E‐09
GO:0008652∼cellular amino acid biosynthetic process	8	13.11	1.63E‐06
GO:0008637∼apoptotic mitochondrial changes	5	11.22	0.000867
GO:0097286∼iron ion import	3	42.62	0.0016
GO:0006749∼glutathione metabolic process	7	5.33	0.0019
GO:0006805∼xenobiotic metabolic process	8	4.37	0.0023
GO:0009612∼response to mechanical stimulus	7	5.06	0.0025
GO:0001558∼regulation of cell growth	8	4.26	0.0027
GO:0018916∼nitrobenzene metabolic process	3	31.96	0.0032
GO:0006534∼cysteine metabolic process	3	31.96	0.0032

**Table 6:** Genes differentially expressed in loss of Y chromosome (LoY) cases for The Cancer Genome Atlas (TCGA) human papillomavirus (HPV)‐positive male head and neck squamous cell carcinoma (HNSCC)

Top 10 most significant gene ontology terms			
Term	No. genes	Fold enrichment	*P* value
**Downregulated genes in LoY cases**			
GO:0006955~immune response	61	3.66	2.92E‐18
GO:0002250~adaptive immune response	36	6.14	4.76E‐18
GO:0050852~T cell receptor signaling pathway	29	4.95	4.26E‐12
GO:0031295~T cell costimulation	21	6.8	1.51E‐11
GO:0050776~regulation of immune response	31	4.4	1.58E‐11
GO:0042110~T cell activation	16	8.6	1.79E‐10
GO:0006954~inflammatory response	44	2.93	5.02E‐10
GO:0007165~signal transduction	89	1.94	2.22E‐09
GO:0042102~positive regulation of T cell proliferation	16	6.73	7.85E‐09
GO:0002407~dendritic cell chemotaxis	9	13.37	1.02E‐07
**Upregulated genes in LoY cases**			
GO:0055114~oxidation‐reduction process	31	2.81	6.78E‐07
GO:0050729~positive regulation of inflammatory response	9	6.61	6.12E‐05
GO:0009636~response to toxic substance	9	5.68	0.000181
GO:0031424~keratinization	7	7.82	0.00025
GO:0035725~sodium ion transmembrane transport	8	5.88	0.000408
GO:0001523~retinoid metabolic process	7	6.16	0.000923
GO:0008544~epidermis development	8	5.05	0.001
GO:0042574~retinal metabolic process	4	17.88	0.0012
GO:0006094~gluconeogenesis	6	7.32	0.0013
GO:0030216~keratinocyte differentiation	7	4.94	0.0029

Redox‐related genes included members of the aldo‐keto reductase (AKR) family 1 (AKR1C1, AKR1C2, and AKR1C3) and G6PD which have previously been linked to resistance to cisplatin‐based chemotherapy and radiotherapy.^24^, ^25^, ^26^ We also observed similar differences in gene expression when male LoY cases were compared with female cases (data not shown).

Genes with immune‐related functions were similarly enriched when gene expression in LoY versus non‐LoY cases was compared in the data reported by Chen et al (Supporting Information Table S1). Although we did not observe a significant enrichment of genes involved in redox processes among genes upregulated in this dataset, we did observe the overexpression of some redox‐related genes, including AKR1C1 and G6PD.

We conclude that LoY in male HNSCC is associated with the reduced expression of immune response genes and the increased expression of genes involved in redox processes and chemotherapy resistance.

### 3.5 LoY is associated with significantly higher levels of autosomal aneuploidy in HPV‐negative male HNSCC

**Figure 4:**
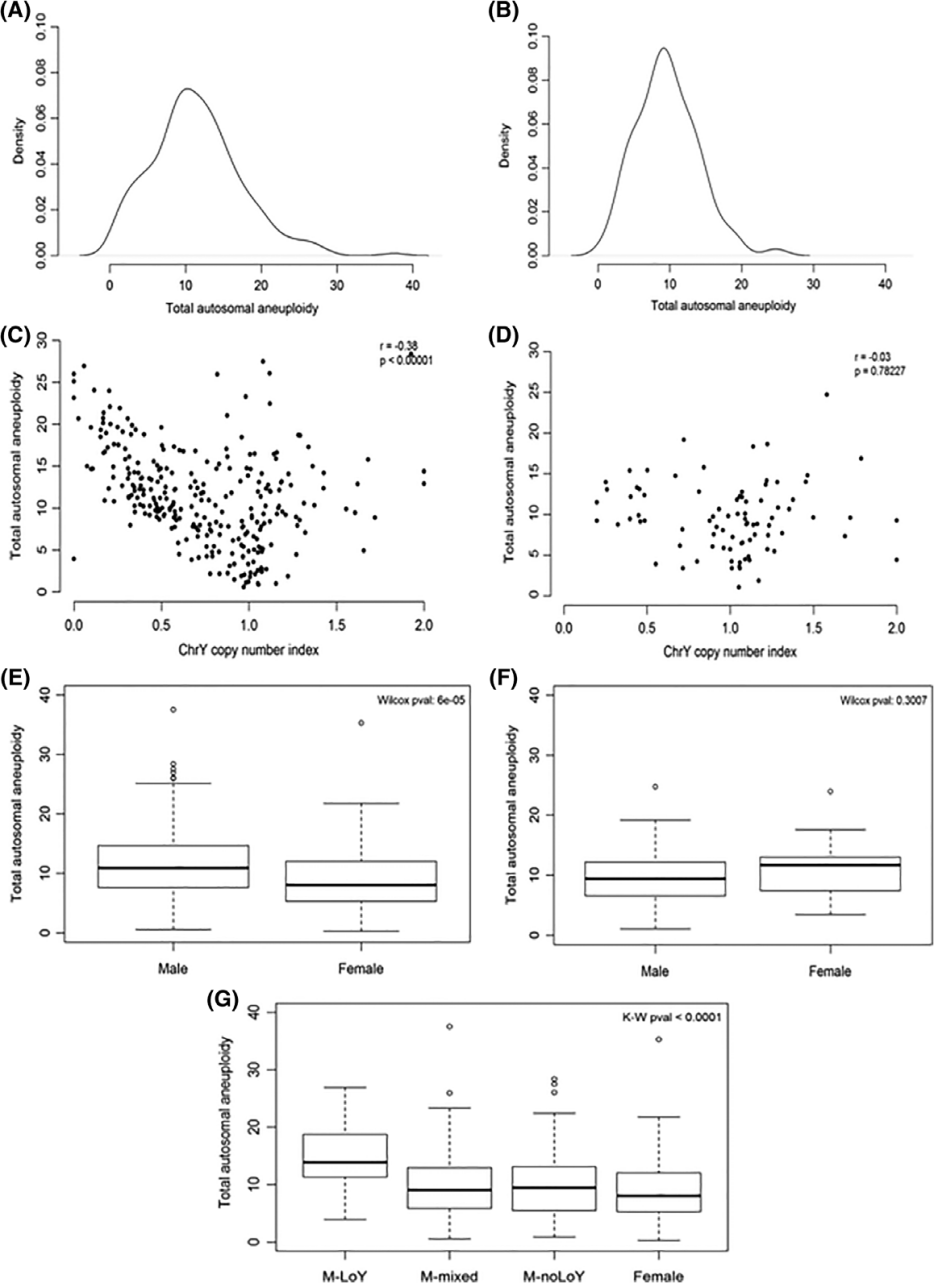
Loss of Y chromosome (LoY) reflects an aneuploid phenotype in human papillomavirus (HPV)‐negative male head and neck squamous cell carcinoma (HNSCC). A,B, Density plots of total autosomal aneuploidy index values for HPV‐negative and HPV‐positive male HNSCC, respectively. C,D, Plots of Y chromosome copy number index values against total autosomal aneuploidy index values for HPV‐negative and HPV‐positive male HNSCC, respectively. E,F, Boxplots of total autosomal aneuploidy index values split by sex for HPV‐negative and HPV‐positive HNSCC, respectively. G, Boxplots of total autosomal aneuploidy index values split by sex for HPV‐negative HNSCC with males split by LoY status

We next considered the possibility that LoY in male HNSCC simply reflects chromosome instability. For each sample, we derived a total (autosomal) aneuploidy index. Unlike the Y chromosome index, the total aneuploidy index did not have a bimodal distribution for either HPV subgroup (Figure 4A,B). Furthermore, there was no significant association between aneuploidy and smoking or age for either HPV subgroup (data not shown).

We found that the Y chromosome index was inversely correlated with the total aneuploidy index, but this was only observed in the HPV‐negative patients (Figure 4C,D). Splitting the HPV‐negative male HNSCC cases as before revealed that LoY cases had a significantly higher total aneuploidy index than non‐LoY cases (data not shown, Wilcoxon *P* < 0.0001), although we also observed that there were some highly aneuploid male tumors with no evidence of LoY.

We next compared aneuploidy levels between male and female HNSCC. We found that the total aneuploidy index for HPV‐negative male HNSCC was significantly higher than for HPV‐negative female HNSCC, but that this was not the case for HPV‐positive HNSCC (Figure 4E,F, Wilcoxon *P* < 0.0001 and *P* = 0.30, respectively). For the HPV‐negative cases, we refined our analysis by splitting the males into those with or without evidence of loss of the Y chromosome (as previously), and also including those cases for which the evidence was uncertain (ie, the samples whose Y chromosome index values fell between the two peaks of the bimodal distribution). We found that the male LoY cases were significantly more aneuploid than not only the other groups of male patients but also the female patients (Figure 4G, Kruskal‐Wallis *P* < 0.0001).

We then refined our analyses by further dividing cases into those which did or did not have evidence of a somatic mutation in the TP53 gene. Somatic mutation data were available for 345 of the 359 male patients (Table 3).

Overall, 244 of 345 patients (70.7%) had evidence of a mutation in TP53. The frequency of mutation was much greater in HPV‐negative cases (223 of 264 = 84.5%) compared with HPV‐positive cases (21 of 81 = 25.9%, Fisher's exact test *P* < 0.0001).

**Figure 5:**
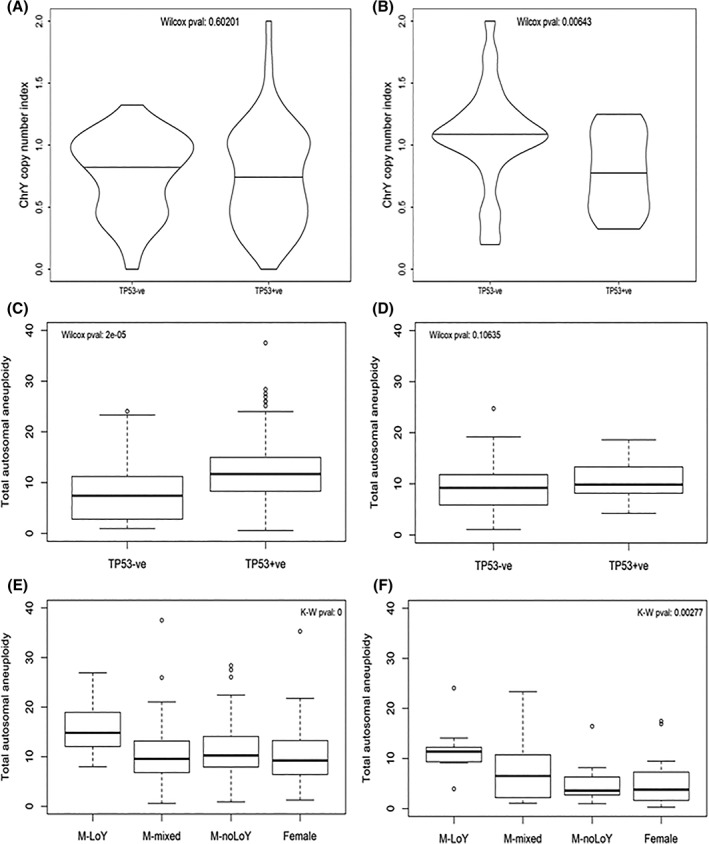
Aneuploidy, but not Loss of Y chromosome (LoY), is associated with TP53 mutation in human papillomavirus (HPV)‐negative male head and neck squamous cell carcinoma (HNSCC). A,B, Violin plots of Y chromosome copy number index values by TP53 mutational status for HPV‐negative and HPV‐positive male HNSCC, respectively—horizontal lines denote median values. C,D, Boxplots of total autosomal aneuploidy index values by TP53 mutational status for HPV‐negative and HPV‐positive male HNSCC, respectively. E,F, Boxplots of total autosomal aneuploidy index values for HPV‐negative HNSCC split by sex, with males further split by LoY status, for cases with or without evidence of a mutation in TP53, respectively

We found that for HPV‐negative male HNSCC, there was no association between the Y chromosome index and evidence of TP53 mutation, whereas for HPV‐positive male HNSCC, there was a statistically significant difference between mutated and nonmutated cases, with the latter having lower levels of LoY (Figure 5A,B, Wilcoxon test *P* = 0.60 and 0.006, respectively). However, in contrast to the LoY results, we found that HPV‐negative male HNSCC with evidence of TP53 mutation was significantly more aneuploid than that without evidence of mutation, whereas the difference for HPV‐positive male HNSCC was not statistically significant (Figure 5C,D, Wilcoxon *P* < 0.0001 and *P* = 0.11, respectively). Finally, we found that for HPV‐negative HNSCC both with and without evidence of TP53 mutation, males with LoY were significantly more aneuploid than other males and also females (Figure 5E,F, Kruskal‐Wallis *P* < 0.0001 and *P* = 0.003, respectively).

We conclude that in HPV‐negative HNSCC, LoY in males is associated with significantly higher aneuploidy compared to both other males and females.

### 3.6 LoY is strongly associated with the overexpression of redox genes implicated in chemotherapy resistance

We next considered the possibility that the differences in gene expression we had observed between LoY and non‐LoY cases were primarily related to autosomal aneuploidy.

First, we ordered cases by total autosomal aneuploidy index and compared global gene expression between the top and bottom thirds. For both HPV‐negative male and female HNSCC, genes downregulated in highly aneuploid cases were significantly enriched for immune‐related genes, and those upregulated were significantly enriched for genes involved in redox processes (Table 7 and Supporting Information Table S2), as we previously described for LoY. Enrichment of immune‐related genes was also observed among genes downregulated in highly aneuploid HPV‐positive male HNSCC (data not shown), but there were too few HPV‐positive female cases for a meaningful comparison.

**Table 7:** Genes differentially expressed in highly aneuploid The Cancer Genome Atlas (TCGA) human papillomavirus (HPV)‐negative male head and neck squamous cell carcinoma (HNSCC)

Top 10 most significant gene ontology terms			
Term	No. genes	Fold enrichment	*P* value
**Downregulated genes in high aneuploidy cases**			
GO:0006955∼immune response	142	5.22	2.03E‐64
GO:0006954∼inflammatory response	111	4.54	2.73E‐43
GO:0060333∼interferon‐gamma‐mediated signaling pathway	41	8.95	2.1E‐29
GO:0007155∼cell adhesion	97	3.27	1.32E‐25
GO:0045087∼innate immune response	92	3.31	1.06E‐24
GO:0002250∼adaptive immune response	49	5.13	9.81E‐22
GO:0060337∼type I interferon signaling pathway	32	7.75	1.73E‐20
GO:0050776∼regulation of immune response	52	4.53	2.52E‐20
GO:0070098∼chemokine‐mediated signaling pathway	33	7.2	6.58E‐20
GO:0006935∼chemotaxis	42	5.33	2.09E‐19
**Upregulated genes in high aneuploidy cases**			
GO:0030855∼epithelial cell differentiation	15	4.65	2.99E‐06
GO:0060070∼canonical Wnt signaling pathway	16	4.18	5.04E‐06
GO:0055114∼oxidation‐reduction process	52	1.91	1.27E‐05
GO:0030326∼embryonic limb morphogenesis	9	4.88	0.000403
GO:0009954∼proximal/distal pattern formation	7	6.33	0.000616
GO:0006749∼glutathione metabolic process	10	3.87	0.00096
GO:0008652∼cellular amino acid biosynthetic process	7	5.84	0.000974
GO:0009952∼anterior/posterior pattern specification	12	3.25	0.0011
GO:0006600∼creatine metabolic process	5	9.86	0.0011
GO:0007224∼smoothened signaling pathway	11	3.46	0.0012

We next split the highly aneuploid male HNSCC into LoY and non‐LoY cases applying the same criteria as were used earlier. For both HPV subgroups, we no longer observed a significant enrichment of immune‐related ontology terms among genes downregulated in LoY cases (data not shown). However, among the highly aneuploid HPV‐negative male HNSCC, we found that genes upregulated in LoY versus non‐LoY cases were still significantly enriched for genes involved in redox processes and the metabolism of chemotherapeutic drugs, again including AKR1C1, AKR1C2, AKR1C3, and G6PD (Table 8).

**Table 8:** Genes upregulated in loss of Y chromosome (LoY) cases among highly aneuploid The Cancer Genome Atlas (TCGA) human papillomavirus (HPV)‐negative male head and neck squamous cell carcinoma (HNSCC)

Top 10 most significant gene ontology terms			
Term	No. genes	Fold enrichment	*P* value
GO:0055114~oxidation–reduction process	30	3.85	9.55E‐10
GO:0043651~linoleic acid metabolic process	5	22.35	5.97E‐05
GO:0006805~xenobiotic metabolic process	8	7.79	7.23E‐05
GO:0044597~daunorubicin metabolic process	4	37.99	0.000118
GO:0044598~doxorubicin metabolic process	4	37.99	0.000118
GO:0006098~pentose‐phosphate shunt	4	27.63	0.000339
GO:0008152~metabolic process	10	4.52	0.000377
GO:0097286~iron ion import	3	75.98	0.000508
GO:0006749~glutathione metabolic process	6	8.14	0.000818
GO:0071395~cellular response to jasmonic acid stimulus	3	56.99	0.001

**Figure 6:**
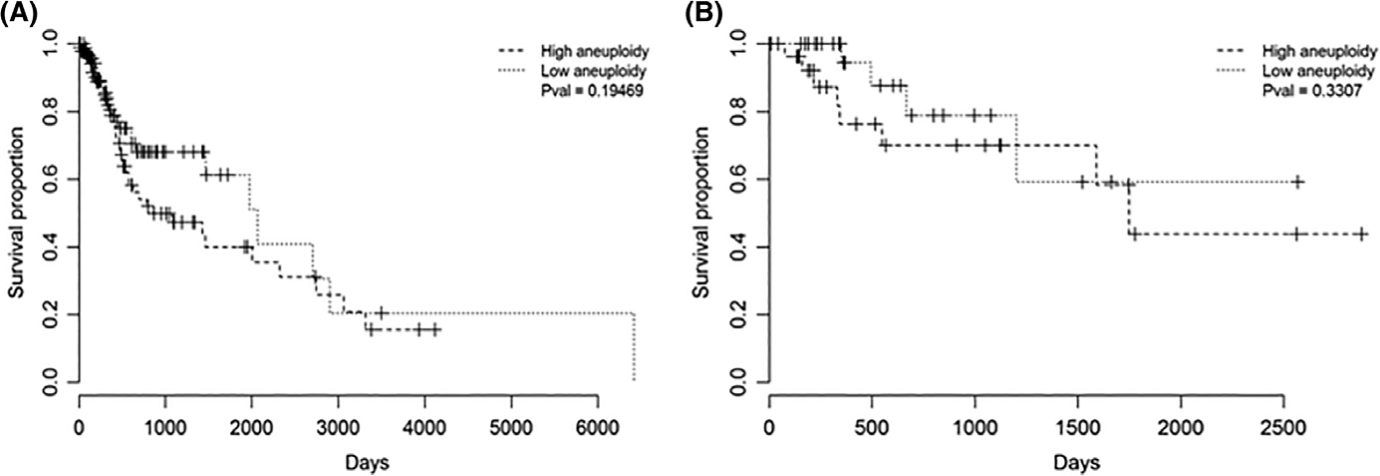
Aneuploidy is not associated with poor survival in male human papillomavirus (HPV)‐negative head and neck squamous cell carcinoma (HNSCC). Kaplan‐Meier plots of overall survival in A, HPV‐negative male HNSCC and B, HPV‐positive male HNSCC, with patients split in each case by low/high total autosomal aneuploidy index

In keeping with these observations, we found no evidence of worse overall survival for highly aneuploid cases (Figure 6, *P* = 0.19 and 0.33 for HPV‐negative and HPV‐positive cases, respectively) and only a modest negative effect in a univariate Cox proportional hazards analysis of all HPV‐negative male patients based on the total aneuploidy index (Wald test *P* = 0.03).

We conclude that in male HPV‐negative HNSCC, LoY is associated with the overexpression of genes involved in redox processes and the metabolism of chemotherapeutic drugs.

## 4 DISCUSSION

Although LoY has previously been documented in male head and neck cancer,^14^ to date its pathogenic significance has been unclear. We took advantage of the large cohort of HNSCC patients with clinical data available from TCGA to explore the prevalence of LoY in HNSCC, and its association with clinicopathological variables, including outcome.

We showed that LoY is evident in around one‐quarter of male head and neck tumors, with higher prevalence in HPV‐negative compared with HPV‐positive tumors, consistent with the idea that these two subcategories have distinct aetiologies.^2^ In our analysis of LoY in the TCGA data, the copy number index rarely reached zero. This is probably explained by the presence of a subset of nonmalignant cells within each sample, as our results reflect the average copy number across all cells, and our adjustment for tumor purity could only make partial correction. Furthermore, there is the possibility that only a subpopulation of tumor cells were affected by LoY. Nonetheless, the FISH analysis, which is able to directly identify LoY, suggested that our estimate of LoY based on TCGA's copy number data closely approximates its true prevalence.

The results of our analysis of corresponding RNA‐seq data from TCGA, and from a separate cohort of male HNSCC, confirmed our findings of Y chromosome loss. In particular, we observed 19 Y‐linked genes whose expression in head and neck tumors was highly inversely correlated with LoY. Previous analysis of the evolution of Y chromosomes in eight different mammalian species identified 12 of these genes (RPS4Y1, ZFY, TBL1Y, PRKY, USP9Y, DDX3Y, UTY, TMSB4Y, NLGN4Y, CYorf15A, KDM5D, and EIF1AY) that are crucial for male viability.^10^ Each of these 12 genes has a homolog on the X chromosome which escapes X‐inactivation, suggesting that they are all dosage‐sensitive. Furthermore, analysis of mutational signatures in over 8200 tumor/normal sample pairs from several different tissue types has suggested that two of these genes, UTY and ZFY, could be tumor suppressor genes.^27^ UTY is of particular interest. Loss of UTY is linked to increased cell proliferation in urothelial bladder cell lines.^28^ Its X‐chromosome homolog (UTX, also known as KDM6A) is a demethylase of lysine 27 on histone 3 (H3K27), which is linked to dysregulated squamous cell differentiation in HNSCC cell lines^29^; UTY may share this enzymatic activity, albeit at a reduced level.^30^ Loss of UTY has also recently been implicated in both myeloid malignancies and pancreatic cancer via demethylase‐independent mechanisms.^31^, ^32^ There is also other experimental evidence to support the tumor suppressive effects of the Y chromosome. For example, reintroducing a lost Y chromosome can suppress tumor formation in a mouse model of prostate cancer,^33^ although this study did not establish the identity of the tumor suppressor gene(s) responsible for this effect.

In keeping with the idea that the Y chromosome has tumor suppressor functions, we observed that HPV‐negative males with LoY have significantly shorter overall survival compared to their counterparts without LoY. Some caution is required when interpreting these data, as the patients had been treated with different regimes. Nonetheless, our findings are consistent with a previous study based on a much smaller cohort of HNSCC which had suggested that LoY was associated with poorer patient outcomes.^14^

Our gene ontology analysis further emphasized the possibility that LoY and non‐LoY male HNSCC represent biologically and clinically distinct subgroups. Of particular relevance was the observation that genes upregulated in LoY cases were significantly enriched for genes involved in redox processes. Disruption of redox homeostasis, resulting in elevated levels of reactive oxygen species in tumor cells has been implicated in the promotion of tumor progression and development of drug resistance.^34^ Genes upregulated in LoY compared with non‐LoY cases included members of the AKRs superfamily of NAD(P)H‐linked oxidoreductases, such as AKR1B10, AKR1C1, AKR1C2, AKR1C3, as well as ALDH3A1, G6PD, GPX2, PIR, SRXN1, and TXNRD1, which are increasingly recognized for their important roles in drug detoxification and xenobiotic metabolism.^24^, ^25^, ^26^, ^35^, ^36^, ^37^, ^38^, ^39^, ^40^, ^41^, ^42^ In particular, the upregulation of AKR1C1, AKR1C2, and G6PD are all associated with resistance to cisplatin‐based chemotherapy in lung cancer,^24^, ^26^ whereas upregulation of AKR1C3 is linked to insensitivity to radiotherapy in oesophageal cancer.^25^ In a recent study of head and neck cancer, tumor redox was associated with poorer patient outcomes, suggesting that its measurement, for example, by 62Cu‐ATSM PET, could be a useful prognostic marker.^43^

As expected, we found a close relationship between LoY and autosomal aneuploidy, especially in patients with HPV‐negative cancer. Indeed, for these cases, autosomal aneuploidy was found to be significantly higher, not only when LoY male HNSCC were compared with non‐LoY male cases, but also when compared to female cases. These observations are consistent with previous findings,^44^, ^45^ and raise the intriguing possibility that LoY may contribute to the initiation of autosomal aneuploidy, potentially representing an initial chromosomal mis‐segregation event that triggers replication stress, DNA damage, and further genome and chromosome instability.^46^ In keeping with the possibility that LoY could be an early event in HNSCC pathogenesis, our analysis of the gene expression reported by Chen et al^16^ identified one oral dysplasia sample with evidence of LoY (data not shown).

Notwithstanding the strong association between LoY and autosomal aneuploidy, there are several reasons why we believe that LoY may have pathogenic significance in its own right. First, a subset of highly aneuploid male HPV‐negative cancers showed no evidence of LoY. Second, while we observed a significant enrichment of redox‐associated genes also in highly aneuploid tumors, a comparison of LoY and non‐LoY cases in this subset revealed even higher levels of redox‐related gene expression among LoY tumors. Thus, LoY apparently superimposes a higher redox state, even among the most aneuploid cancers. Third, while LoY was strongly associated with poorer overall survival in men with HPV‐negative HNSCC, we found less compelling evidence of worse survival for highly aneuploid cases.

Previous work suggests that UTY, and KDM5D, which is downregulated upon LoY, can epigenetically regulate innate and adaptive immune responses.^47^, ^48^ Furthermore, in Drosophila, it has been shown that large regions of heterochromatin within the Y chromosome may serve as genome‐wide regulators of biological functions, including immunity.^49^ Therefore, we were intrigued by the observation that LoY was associated with decreased expression of immune‐related genes. However, when highly aneuploid male HNSCC were split by LoY status, we no longer observed any enrichment of immune‐related ontology terms among genes downregulated in LoY cases. Thus, the downregulation of genes with immune‐related functions appears to be primarily driven by the strong association with aneuploidy. Consistent with this, we also observed a strong association between aneuploidy and the reduced expression of immune response genes in female HNSCC. These observations are in keeping with the findings of Davoli et al, who showed that across 12 human cancers, including HNSCC, highly aneuploid tumors are depleted for the expression of markers of cytotoxic infiltrating immune cells, especially CD8+ T cells.^50^ Furthermore, their analysis of data from two clinical trials of immune checkpoint blockade therapy in metastatic melanoma revealed that tumor aneuploidy inversely correlated with patient survival. Therapeutic targeting of tumor immune suppression in HNSCC is an active area of research, as only a small subset of tumors responds to immune checkpoint inhibition.^51^ It remains to be seen if LoY could be useful in identifying aneuploid HNSCC and hence tumors that are less likely to respond to immune checkpoint inhibitors.

Previous research has suggested that LoY is associated with smoking.^13^ We found a borderline association among patients with HPV‐negative tumors, although we were unable to account for the effects of alcohol consumption as data were unavailable for over half of the patients. Our observation that LoY was less frequent among patients who had stopped smoking compared to current smokers suggests that the effects of smoking on LoY are potentially reversible, which is consistent with previous research.^13^ We found no significant association between age and LoY which is also consistent with a previous report.^52^

In summary, our analysis revealed that LoY is a common structural chromosomal abnormality in male HNSCC. Although LoY is associated with aneuploidy, the strong association between LoY and impaired survival linked to the overexpression of chemotherapy resistance genes suggests that LoY has distinct biological and clinical significance that warrants further investigation.

## Supporting information

Supporting Information Table S1 Genes differentially expressed in LoY cases from Chen et al.

Supporting Information Table S2 Genes differentially expressed in highly aneuploid HPV‐ve female HNSCC
